# Association between refrigerator use and the risk of gastric cancer: A systematic review and meta-analysis of observational studies

**DOI:** 10.1371/journal.pone.0203120

**Published:** 2018-08-30

**Authors:** Shijiao Yan, Yong Gan, Xingyue Song, Yunqiang Chen, Na Liao, Song Chen, Chuanzhu Lv

**Affiliations:** 1 School of International Education, Hainan Medical University, Haikou, Hainan, P. R. China; 2 Department of Social Medicine and Health Management, School of Public Health, Tongji Medical College, Huazhong University of Science and Technology, Wuhan, Hubei, P. R. China; 3 Emergency and Trauma College, Hainan Medical University, Haikou, Hainan, P. R. China; 4 Department of Nursing, the Second Affiliated Hospital of Hainan Medical University, Haikou, Hainan, P. R. China; 5 Department of Emergency, the First Affiliated Hospital of Hainan Medical University, Haikou, Hainan, P. R. China; University Hospital Llandough, UNITED KINGDOM

## Abstract

There were many observational studies that examined the association between refrigerator use and stomach cancer. However, the results remain to be a contradiction. This study aimed to evaluate the association between refrigerator use and the risk of gastric cancer. We systematically searched the PubMed, Embase, Web of Science databases (up to 31 May 2017), and manually reviewed the references lists of retrieved articles, to identify studies that evaluated the association between refrigerator use and the risk of gastric cancer. Observational studies reporting odds ratio (OR) with 95% confidence intervals (CIs) for the relationship between refrigerator use and the risk of gastric cancer were included. Two authors independently reviewed and selected eligible studies and conducted the study quality evaluation. We included a total of twelve studies enrolling 14,361 individuals. The summarized OR the association between refrigerator use and the risk of gastric cancer was 0.70 (95% CI, 0.56–0.88; *P<*0.001). Subgroup analysis showed that a significantly inverse association between refrigerator use and gastric cancer risk was observed in in some Asian countries (OR = 0.68, 95% CI, 0.50–0.93; *P =* 0.002), but not in some Western countries, such as Germany, etc. Refrigerator use is significantly associated with a decreased risk of gastric cancer. Further studies are warranted to confirm whether refrigerator use could reduce the risk of gastric cancer among some Asian countries.

## Introduction

Gastric cancer is the fourth most frequently diagnosed cancer worldwide, behind lung, breast as well as colorectal cancers, and also the globally the second common cause of cancer death [1]. Approximately 951,600 new stomach cancer cases and 723,100 stomach cancer-oriented deaths have happened in 2012. Eastern Asia and Europe, South America embraced the highest morbidity of gastric cancer [2]. Thus, it is of great public health importance to identify the modifiable risk factors for the primary prevention of gastric cancer.

There are increasing number of people and surveys paying close attention to the entanglement between refrigerator use and the risk of gastric cancer, which has been of considerable interest in this field since last early periods of 1980s, when a case-control research proposed by Howson and his colleagues suggested that refrigerator use may play a protective role against gastric cancer [3]. Increasing interests have been received by the public about the effect of refrigerator use on the risk of gastric cancer despite the findings from several studies on epidemiology that have investigated the association between refrigerator use and the risk of gastric cancer with inconsistent results. Therefore, it was necessary to summarize the epidemiological evidence to date on the link between refrigerator use and the risk of gastric cancer. Meta-analysis was a powerful instrument to establish the relationships between exposures and health outcomes due to the fact it is in view of a greater range of participants, a larger sample size, and more cases than any individual study. Thus, a meta-analysis of observational studies was conducted to examine whether refrigerator use does a protective factor against gastric cancer.

## Materials and methods

The meta-analysis was reported according to the checklists of Meta-Analysis of observation in Epidemiology (MOOSE) instructions for its background, design, analysis, and interpretation [4].

### Study strategy

We did a thorough search the PubMed, Embase, and Web of Science databases till May 2017 for studies that investigated the association between refrigerator use and the risk of gastric cancer. The search terms were “refrigerator” or “refrigerator use” or “fridge use” or “freezer use” or “dietary intake” and “gastric” or “stomach” and “carcinoma” or “neoplasm” or “cancer” or “tumor”. The search was limited to studies in humans without any restrictions on language. Additionally, the reference lists of the retrieved articles were manually reviewed. The titles and abstracts of all identified articles were initially screened by one investigator (N.L.) for potentially relevant articles, and the eligibility of the selected full-texts articles was reviewed by two independent investigators (S.J.Y. and Y.G.).

### Inclusion criteria

Studies that met the following criteria were included in the meta-analysis: (1) be was a case-control or cohort study design, (2) the exposure of interest was refrigerator use and the outcome of interest was gastric cancer, (3) the study reported the risk estimates with 95% confidence intervals (CIs) for the relationship between refrigerator use and the risk of gastric cancer or provided sufficient information for their calculation. Animal cases, clinical experiments, reviews, letters, and commentaries were excluded. Once the study populations were stated twice or above, we included the publication that with largest number of cases or those presented results with most complete information.

### Data extraction

We extracted the following data from the included studies: the first author’s name, year of publication, study site, study design, sample size, participants’ age range or average age at entry, follow-up (for cohort study) time period, outcome measurement, number of case and control group, number of refrigerator users, risk estimates and its 95% CI, and covariates adjusted for in statistical analysis. Two investigators (S.J.Y. and. S.C.) independently extracted the data.

### Quality assessment

Two investigators (X.Y.S. and Y.Q.C.) independently performed the quality assessment via the Newcastle-Ottawa Scale (NOS) (for cohort and case-control study) [5].The scale was a nine-point that distributes points based on the selection of the cohort study or case-control (0–4 points), the comparability (0–2 points), and the determination of the demonstration or the outcomes of targeted participants (0–3 points). The scores of 0–3, 4–6, and 7–9 were defined as the low, moderate, and high quality of studies, respectively.

### Data synthesis and statistical analysis

We prioritized the multivariable adjusted risk estimates at which they were reported. The odds ratios (ORs) were known to be a normally present measure for the relationship of refrigerator use with the risk of gastric cancer. We integrated the case-control and cohort research primarily in this meta-analysis, because ORs and relative risks (RRs) provide similar estimates of risk in the case of rare outcome [6]. In studies reporting results separately by duration of refrigerator use, we combined the estimation through the fixed-effects model for an overall estimate for gastric cancer in the primary meta-analysis [7].

Statistical heterogeneity across studies was evaluated with the *I*^*2*^ statistic, where values of 25%, 50% and 75% represented cut-off points for low, moderate and high degrees of heterogeneity, respectively [8]. We used the DerSimonian-Laird inverse-variance-weighted random-effects model to pool results across studies if there was a moderate and above heterogeneity, otherwise the fixed-effects model was used. We conducted subgroup analyses to explore the potential heterogeneity across studies, and meta-regression analysis was used to examine the differences among subgroups (using STATA ‘metareg’ command). Sensitivity analysis was performed with removing one study at a time [9]. Potential publication bias was assessed with the funnel plot and the Egger’s linear regression test [10], with results that publication bias indicated at *P*< 0.10. And all statistical analyses were conducted using STATA version 12.0 (Stata Corp, College Station, TX). Except where otherwise specified, *P* values were two-tailed significantly with level at 0.05.

## Results

### Study selection

**Fig 1:**
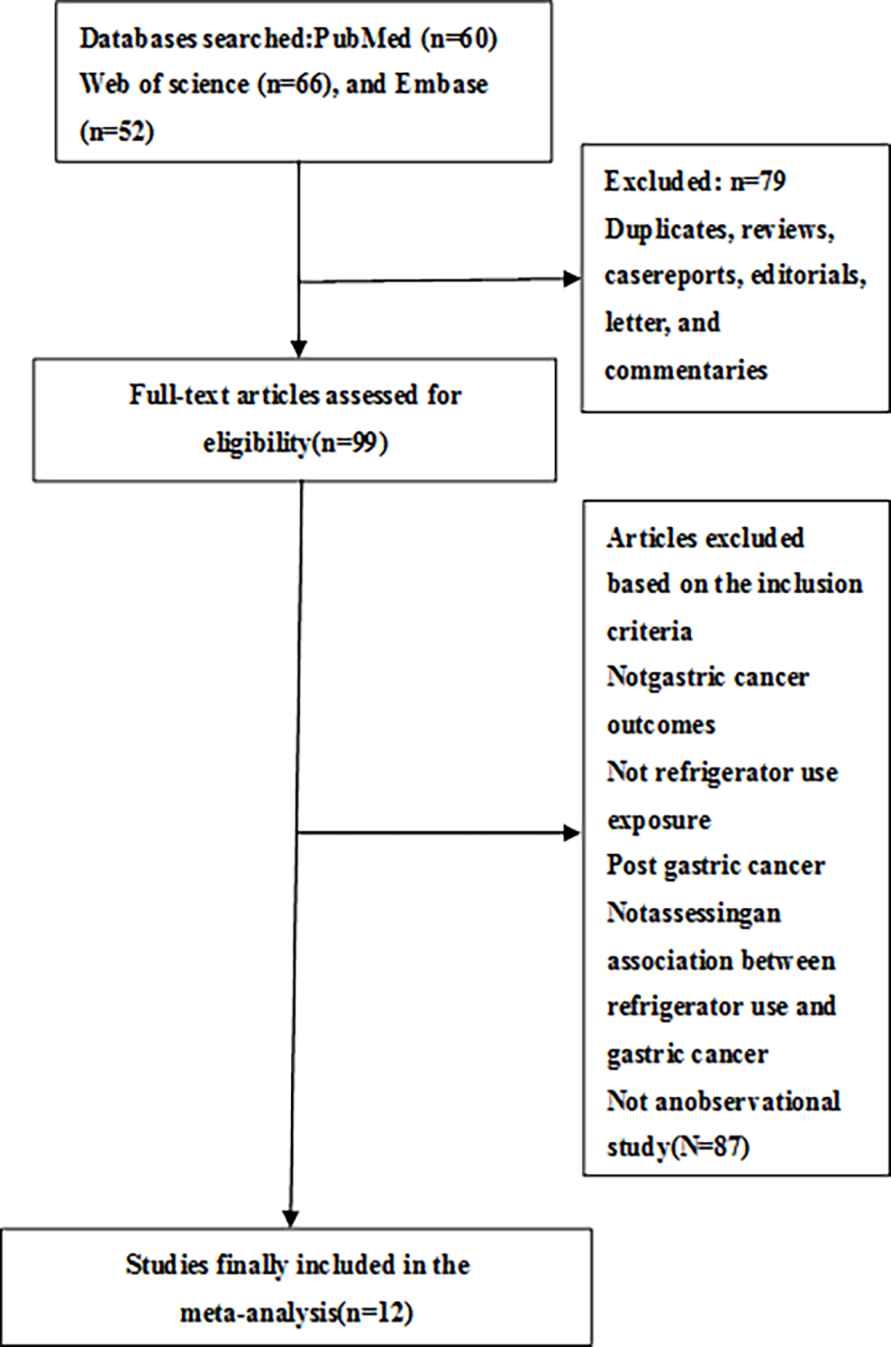
Study selection process for the Meta-analysis.

The results of literature retrieval and research selection are shown in Fig 1. Initial 178 articles were searched from the PubMed, Embase, and Web of Science databases. According to the preliminary screening of titles and abstracts, 99 articles were further evaluated in full text. After retrieving the full-text review for detailed evaluation, 12 studies assessing the relationship between the use of refrigerators and the risk of gastric cancer were identified [11–22].

### Study characteristics

Main characteristics of those included studies published between 1990 and 2011 are presented in Table 1, including one prospective cohort study [22], and eleven case-control studies [11–21].Seven studies were from Europe [11, 12, 15–17, 21, 22], one from the Venezuela [19], and four from Asia [13, 14, 18, 20]. Participant number in each study ranged from 376 to 3,405 with a total of 14,361 individuals. The number of gastric cancer cases ranged from 143 to 746, with a total of 3,987 reported gastric cancer cases. The major adjustment confounding factors covering age, gender, smoking status, body mass index (BMI), and socio-economic status. The average NOS score for all studies was 7.8.

**Table 1:** Characteristics of studies included in the meta-analysis of refrigerator use in relation to risk of gastric cancer.

Source	Design and study location	Sex	Age at baseline, years*	No. of participants	No. of case	No. of control/cohort size		No. of refrigerator usage	Outcome ascertainment	Adjustment for cofounders		Quality assessment	
Munoz et al., 2001	Case-control, Venezuela	F/M	Case: 35+, Control: 35±5	777	292		485	Case: 282; Control: 466	Histologically confirmed		Age, sex and SES		9
La Vecchia, et al. 1990	Case-control, Italy	F/M	Case: 27–74; Control: 25–74	1749	526		1223	NR	Histologically confirmed		Age, sex, area of residence, education, and selected indicator foods (pasta or rice, maize, green vegetables and fresh fruit).		6
Fei, et al., 2006	Case-control, China	F/M	Case: 29–91; Control: 28–93	756	189		567	Case: 173; Control: 557	Histologically confirmed		Income, having three meals at regular time, Taking meals slowly, hot food, fried food, fresh vegetables, fresh fruits, milk products, animal red meat, drinking green tea, vitamins, past history of gastric diseases, and family history of cancer		7
Hansson, et al., 1993	Case-control, Sweden	F/M	40–79	1017	338		679	NR	Newly diagnosed and histologically confirmed		Age, sex, SES, BMI, vegetable and fruit consumption		9
Peleteiro, et al., 2011	Case-control, Portugal	F/M	18–92	1071	422		649	NR	Medical records		Age, sex, education, smoking and Helicobacter pylori infection		9
Pakseresht, et al., 2011	Case-control, Iran	F/M	Case: 66.3 (11.3), Control: 62.9 (11.1)	590	286		304	Case: 275; Control: 301	Histologically confirmed		Age, sex, education, living area, smoking gastric symptoms, income, owning refrigerator, seeds preparing method, frying, H.P infection and total energy intake		9
Binici, et al., 2009	Case-control, Turkey	F/M	Case: 21–92; Control: 30–91	376	188		188	Case: 70; Control: 137	Histologically confirmed		Unadjusted		8
Cai, et al., 2003	Case-control, China	F/M	30–79	603	381		222	Case: Cardia: 83; Non-cardia: 104; Control: 139	Histologically confirmed		Age, sex, smoking, drinking, and family cancer history in the first–degree relatives		7
Van den Brandtt, et al.,2003	Cohort, Netherlands	F/M	55–69	3405	282		3123/120852	Case:181; Control: 1952	Histologically confirmed		Age, sex, smoking status, level of education, stomach disorders and stomach cancer in the family		9
La Vecchia, et al.,1995	Case-control, Italy	F/M	Case: 19–74 Control: 19–74	2799	746		2053	Case: use of refrigerator <30 years vs. 30+ years (380 vs.366); Control: use of refrigerator <30 years vs. 30+ years (1023 vs.1030)	Histologically confirmed		Age, sex, education, traditional foods, Beta-carotene, vitamin C intake, and family history of stomach cancer		7
Boeing, et al.,1991	Case-control, Germany	F/M	32–80	722	143		579	Case: use of refrigerator <24 years, 25–29, 30+ years (41 vs. 37 vs.58); Control: use of refrigerator <24 years, 25–29, 30+ years (127 vs. 159 vs.281)	Histologically confirmed		Age, sex, hospital, water supply, and smoking with meant at home,		7
Mathew, et al., 2000	Nested case-control, India	F/M	20+	499	194		305	Case:106; Control: 188	Histologically confirmed		Age, sex, religion, education, smoking and alcohol habits		7

*Mean or median duration of follow-up.

Abbreviations: BMI, body mass index; NR; not report; SES, socio-economic status.

### Association between refrigerator use and the risk of gastric cancer

**Fig 2:**
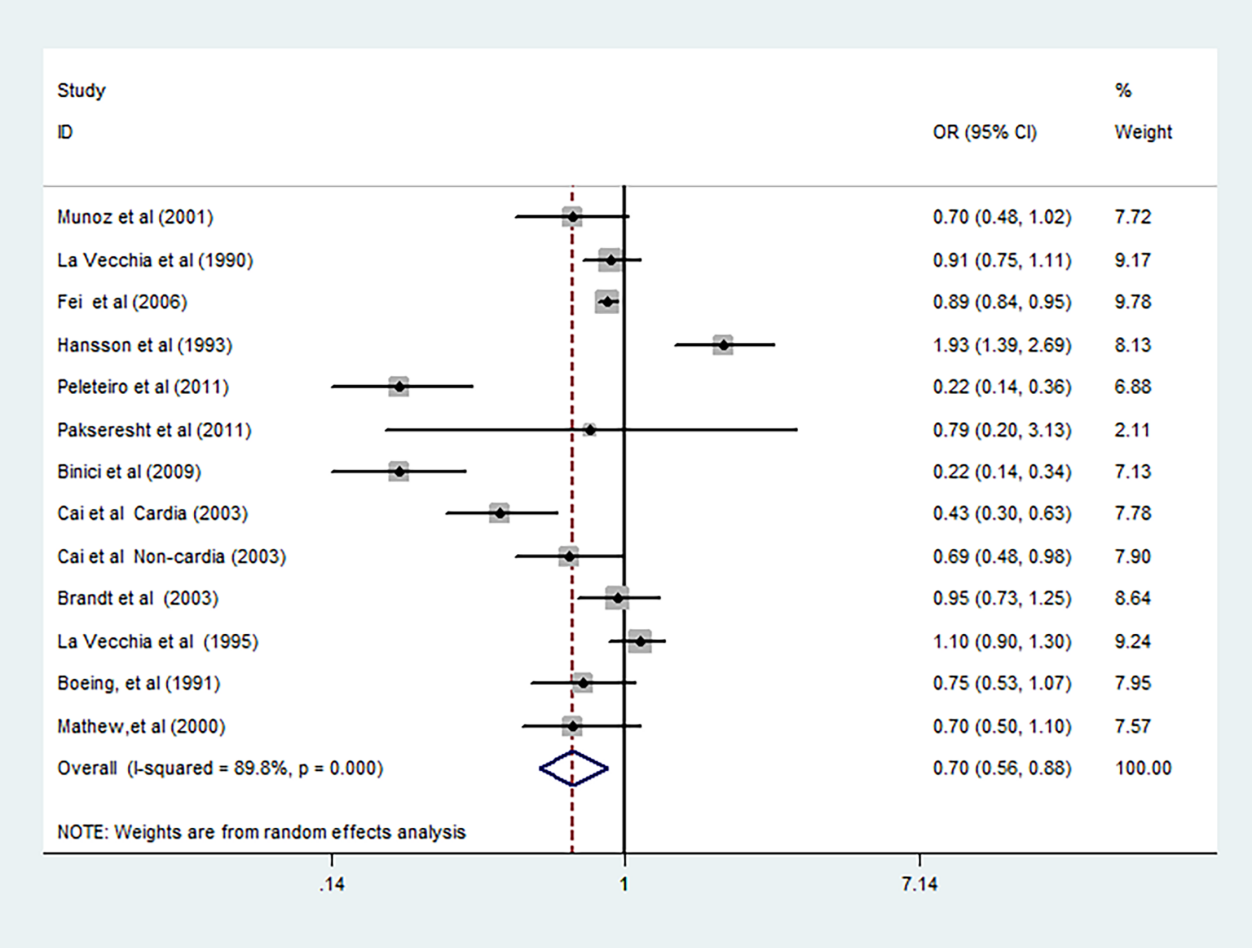
The summarized random-effects ORs and 95%CIs for the association of refrigerator use and risk of gastric cancer.

The random-effects model results with the ORs of gastric cancer in relation to ever use of refrigerator are shown in Fig 2. Five reports from 4 studies suggested an inverse relationship between refrigerator use and the risk of gastric cancer. The summarized OR of gastric cancer for refrigerator use was 0.70 (95% CI, 0.56–0.88) with a high heterogeneity (*P*<0.001; *I*^*2*^ = 89.80%).

### Subgroup analyses

Table 2 shows the results of subgroup analysis of the stability of the major findings, and explored the sources of potential heterogeneity. We conducted subgroup analyses by study location, study quality, and whether age, smoking, BMI, socio-economic status, or family history of gastric cancer were controlled in models in order to assess the impact of specific study characteristics on the association of the risk of gastric cancer with use of refrigerator. Refrigerator use was connected with a decreased risk of gastric cancer in major subgroups. The risk was more obviously decreased in some Asian countries and statistical control for age or smoking. Subgroup analyses by study location showed that a significantly inverse association between the risk of gastric cancer and refrigerator use was identified in some Asian countries, but a significant association was not found in some European and American countries, such as Italy, Germany, and the Netherlands, etc. (see Table 2).

**Table 2:** Subgroup analyses of odds ratio (OR) of gastric cancer according to refrigerator use*.

	No. of reports	*OR*	(95%CI)	*I*^*2*^	*P* for heterogeneity	*P* value for interaction
**Location**						
**European countries**	7	0.70	0.45 to 1.08	94.00%	<0.001	0.10
Italy	2	1.00	0.83 to 1.21	47.70%	0.167	
Sweden	1	1.93	1.39 to 2.68	NA	NA	
Netherlands	1	0.95	0.73 to 1.24	NA	NA	
Germany	1	0.75	0.53 to 1.07	NA	NA	
Turkey	1	**0.22**	**0.14 to 0.34**	NA	NA	
Portugal	1	**0.22**	**0.14 to 0.36**	NA	NA	
**American countries**	1	0.70	0.48 to 1.02	NA	NA	
Venezuela	1	0.70	0.48 to 1.02	NA	NA	
**Asian countries**	5	**0.68**	**0.50 to 0.93**	76.60%	0.002	
China	3	0.75	0.55 to 0.96	49.34%	0.034	
India	1	0.70	0.50 to 1.10	NA	NA	
Iran	1	0.79	0.23 to 3.13	NA	NA	
**Study quality**						
Score>7	6	0.60	0.29 to 1.23	94.40%	<0.001	0.53
Score≤7	7	**0.80**	**0.68 to 0.94**	75.10%	<0.001	
**Publication year**						
Before 2000	4	**1.09**	0.80 to 1.49	84.1%	<0.001	0.01
2000–2011	9	**0.55**	**0.39 to 0.77**	90.6%	<0.001	
**Controlling age in models**						
Yes	11	**0.75**	**0.57 to 0.99**	87.60%	<0.001	0.33
No	2	0.45	0.11 to 1.77	97.30%	<0.001	
**Controlling smoking in models**						
Yes	7	**0.59**	**0.41 to 0.84**	82.10%	<0.001	0.35
No	6	0.84	0.62 to 1.13	92.40%	<0.001	
**Controlling BMI in models**						
Yes	1	**1.93**	**1.39 to 2.68**	NA	NA	0.07
No	12	**0.65**	**0.52 to 0.81**	88.40%	<0.001	
**Controlling SES in models**						
Yes	8	0.83	0.63 to 1.09	88.90%	<0.001	0.28
No	5	**0.55**	**0.35 to 0.87**	89.90%	<0.001	
**Controlling family history of gastric cancer in models**						
Yes	3	**0.59**	**0.43 to 0.81**	89.80%	<0.001	0.64
No	10	**0.74**	**0.57 to 0.96**	91.10%	<0.001	

Abbreviations: BMI, body mass index; OR, odds ratio; SES, socio-economic status.

*The ORs were summarized by using random-effects meta-analysis.

### Sensitivity analyses

Sensitivity analysis was used to discover the potential sources of heterogeneity between the use of refrigerators and the risk of stomach cancer, and to check the effects of various exclusions on the associated OR, and test the robustness of all of the above results. We compared the fixed and the random-effects models, but found no significant difference in the summarized OR between the two (fixed-effects model summarized OR = 0.87, 95% CI: 0.82–0.91, random-effects model summarized OR = 0.70, 95% CI: 0.56–0.88). Restricting analysis to restricting analysis to studies that reported the association between refrigerator use and the risk of gastric cancer with a reference group for never refrigerator uses (OR = 0.56; 95% CI: 0.36–0.87; *I*^*2*^ = 85.8%) did not alter the primary results. Reverse correlation has no substantial change in leave-one-out analyses omitting a study at each turn, a summarized OR ranging from 0.65 (95% CI, 0.52–0.81; *P* = 0.147) to 0.78 (95% CI, 0.64–0.95; *P*<0.001), which indicated that the overall results were not significantly affected by any individual study.

### Publication bias

**Fig 3:**
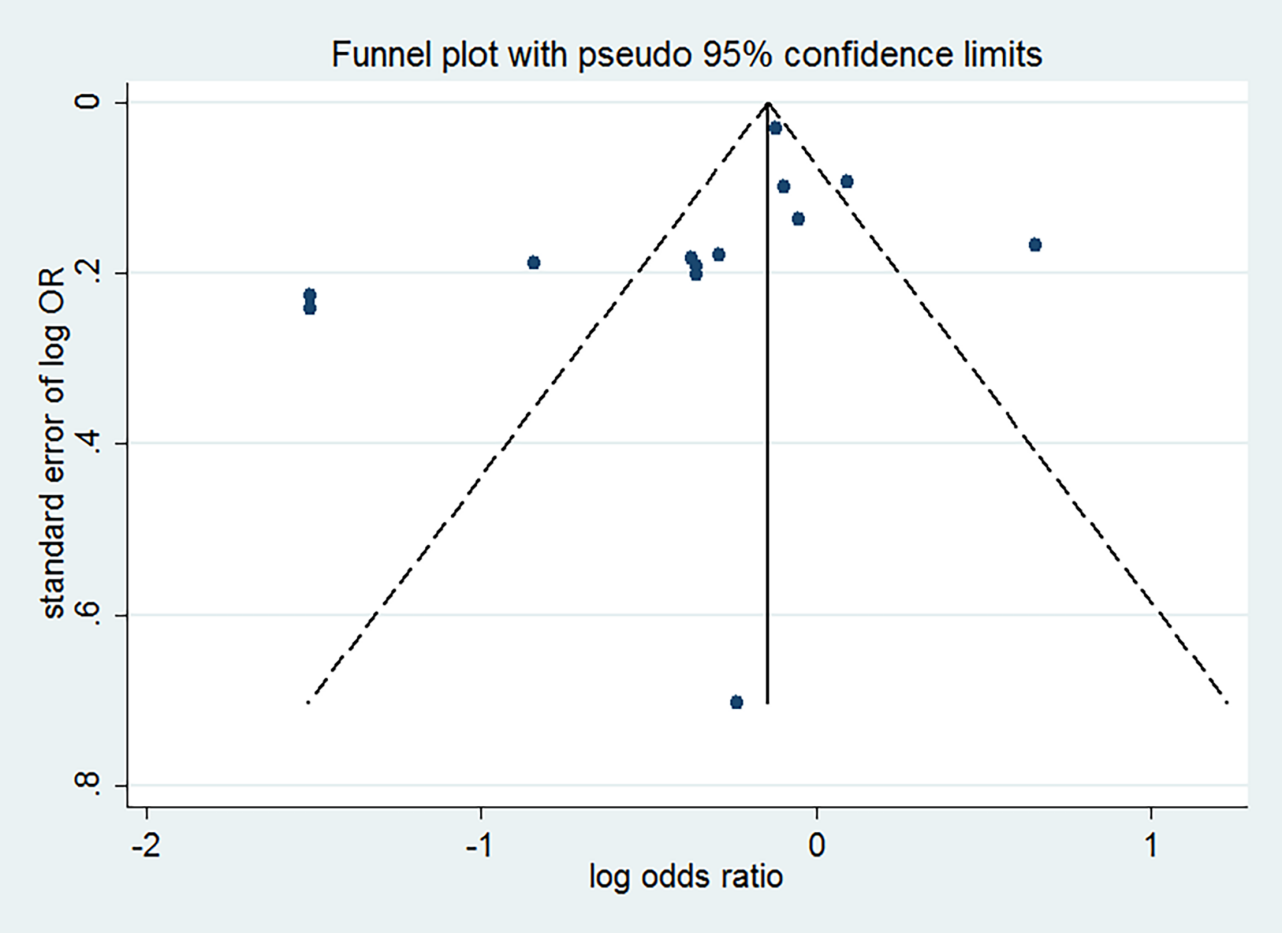
Funnel plot for studies of refrigerator use in relation to gastric cancer.

No visual asymmetry was found in the visual examination of funnel plots (see Fig 3). The Egger’s tests did not provide any important evidence about publication bias for studies that investigated the relationship of refrigerator use with gastric cancer (Egger’s *P* = 0.183).

## Discussion

Refrigerator use was inversely associated with gastric cancer based on data from 12 observations with 13 separate reports with 14,361 individuals. Compared with individuals who had never or less exposure to refrigerator use, the risk of gastric cancer was decreased by 30%.

We obtained a valuable and important finding in subgroup analyses. We found that a significantly negative correlation between the use of refrigerators and the risk of gastric cancer in some Asian countries (OR = 0.68; 95% CI, 0.50 to 0.93), but not in the some Western (European and American) countries, which was an interesting occurrence. This difference may in part reflect the difference in the prevalence of Helicobacter pylori (H. pylori) infection between European and Asian, which was significant risk factor for gastric cancer [23–25]. The null inverse association in some Western countries might be driven by the study by Hasson et al [15]. When we excluded this study, the inverse association between refrigerator use and gastric cancer in some European countries became statistically significant (OR = 0.59; 95%CI: 0.38–0.93). Thus, the differences need to be further investigated.

Certain potential biological mechanisms may explain the links between refrigerator use and the risk of gastric cancer. First, refrigerator could make foods and vegetables stay fresh for longer time, and reduce the likelihood of producing nitroso compounds [26], which has been established to be a risk factor for gastric cancer [27, 28]. Second, refrigeration may keep vitamins as well as other antioxidant at a higher level, which in turn protect the individuals from exposure to nitroso compounds and other carcinogens [13, 29]. Third, the usage of refrigerator could reduce the need for and use of traditional preserving methods such as salting, smoking, pickling, and curing, which also could lead to cancer [3, 30–33].

Our study has several notable strengths. This is the first systematic and quantified meta-analysis of studying the relationship between the use of refrigerators and the risk of gastric cancer. Because of the widespread use of refrigerator worldwide, our results are of great interests to both medical science and the public. Second, according to our subgroup analysis, there was a significantly negative relationship between the use of refrigerator and the risk of gastric cancer in some Asian countries, which provided a new insight for future research of how the biological mechanisms of refrigerator use and gastric cancer are affected by ethnicity.

Some potential limitations to this study should be taken into consideration. First, most of the original studies used in our meta-analysis are of case-control study design, which is particularly vulnerable to potential biases (including selection bias and information bias). Second, the included studies were conducted in different populations, and this may confuse our analysis of the special association between the use of refrigerator and the risk of gastric cancer. Third, the different definitions for refrigerator usage across studies may bring the heterogeneity into studies’ results. Final, there was the evidence of heterogeneity across studies used for the analysis of association between refrigerator use and the risk of gastric cancer. The heterogeneity might result from the difference of participants’ characteristic, sample sizes, exposure definitions, and study designs. Thus, the results of this meta-analysis should be interpreted cautiously.

More effort should be put into future research. First, the mostly research were from Asia and Europe, and only one in Venezuela. In view of the differences in potential disease impacts among different geographical locations and races, it may provide more information if there were data from more areas (such as Africa).

## Conclusions

Our meta-analysis indicates that the risk of gastric cancer is 30% lower among refrigerator users. Subgroup analyses suggest that a use of refrigerator is inversely associated with the risk of gastric cancer in some Asian countries. Studies with more samples and longer follow-up times are warranted to replicate our results.

## Supporting information

S1 Checklist PRISMA 2009 checklist. (DOC)

## Abbreviations

BMI: Body mass index
OR: Odds ratio
CI: Confidence intervals
